# Trinucleotide Repeats: A Structural Perspective

**DOI:** 10.3389/fneur.2013.00076

**Published:** 2013-06-20

**Authors:** Bruno Almeida, Sara Fernandes, Isabel A. Abreu, Sandra Macedo-Ribeiro

**Affiliations:** ^1^Instituto de Biologia Molecular e Celular, Universidade do Porto, Porto, Portugal

**Keywords:** amino acid-repeats, microsatellites, protein complexes, protein aggregation, amyloid, protein structure

## Abstract

Trinucleotide repeat (TNR) expansions are present in a wide range of genes involved in several neurological disorders, being directly involved in the molecular mechanisms underlying pathogenesis through modulation of gene expression and/or the function of the RNA or protein it encodes. Structural and functional information on the role of TNR sequences in RNA and protein is crucial to understand the effect of TNR expansions in neurodegeneration. Therefore, this review intends to provide to the reader a structural and functional view of TNR and encoded homopeptide expansions, with a particular emphasis on polyQ expansions and its role at inducing the self-assembly, aggregation and functional alterations of the carrier protein, which culminates in neuronal toxicity and cell death. Detail will be given to the Machado-Joseph Disease-causative and polyQ-containing protein, ataxin-3, providing clues for the impact of polyQ expansion and its flanking regions in the modulation of ataxin-3 molecular interactions, function, and aggregation.

## Trinucleotide Repeats and Human Disease

**Figure 1:**
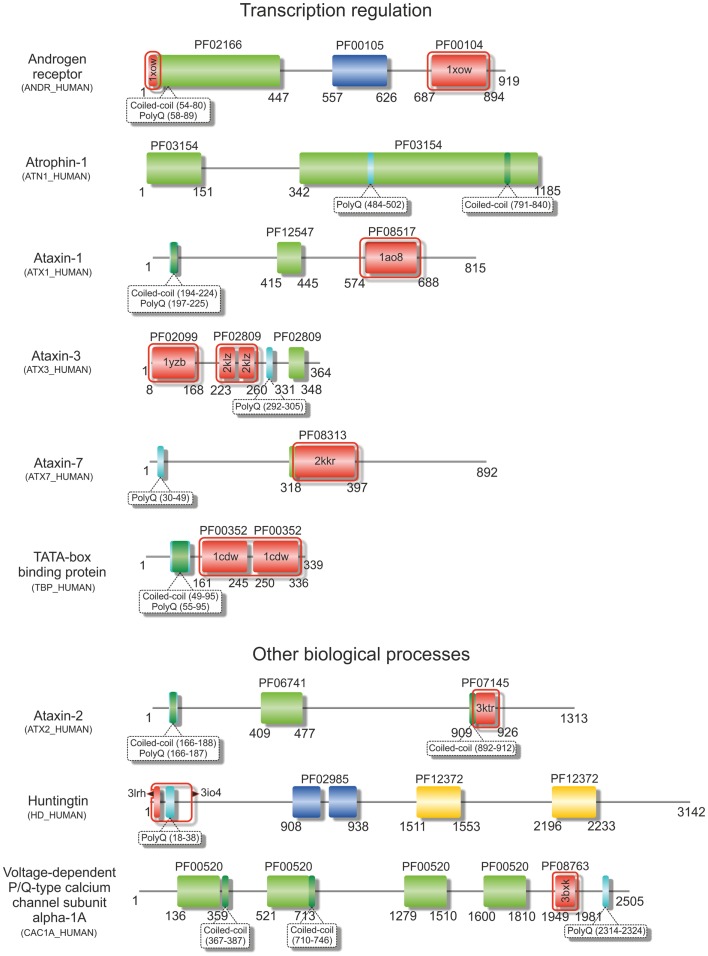
**Structural variability of proteins encoded by TNR-containing genes**. Illustrative domain graphics of the multi-domain structure of proteins associated with polyQ-expansion diseases. All proteins shown are referenced by their name as annotated in UniProt. The protein domains for which information is annotated in the Pfam database are shown as colored boxes with Pfam family accession code referenced above the domain box. Complete names of domains can be assessed by searching the specific Pfam accession code at http://pfam.sanger.ac.uk/. Numbers below the domain schemes represent amino acid residue numbers. Regions containing the amino acid repeats and with a prediction for formation of coiled-coils (as annotated in UniProt) are shown as well as regions with known 3D structure (boxed in red, with PDB accession codes shown). Notice the predominant location of the repeat regions within the N-terminal regions of the proteins.

**Figure 2:**
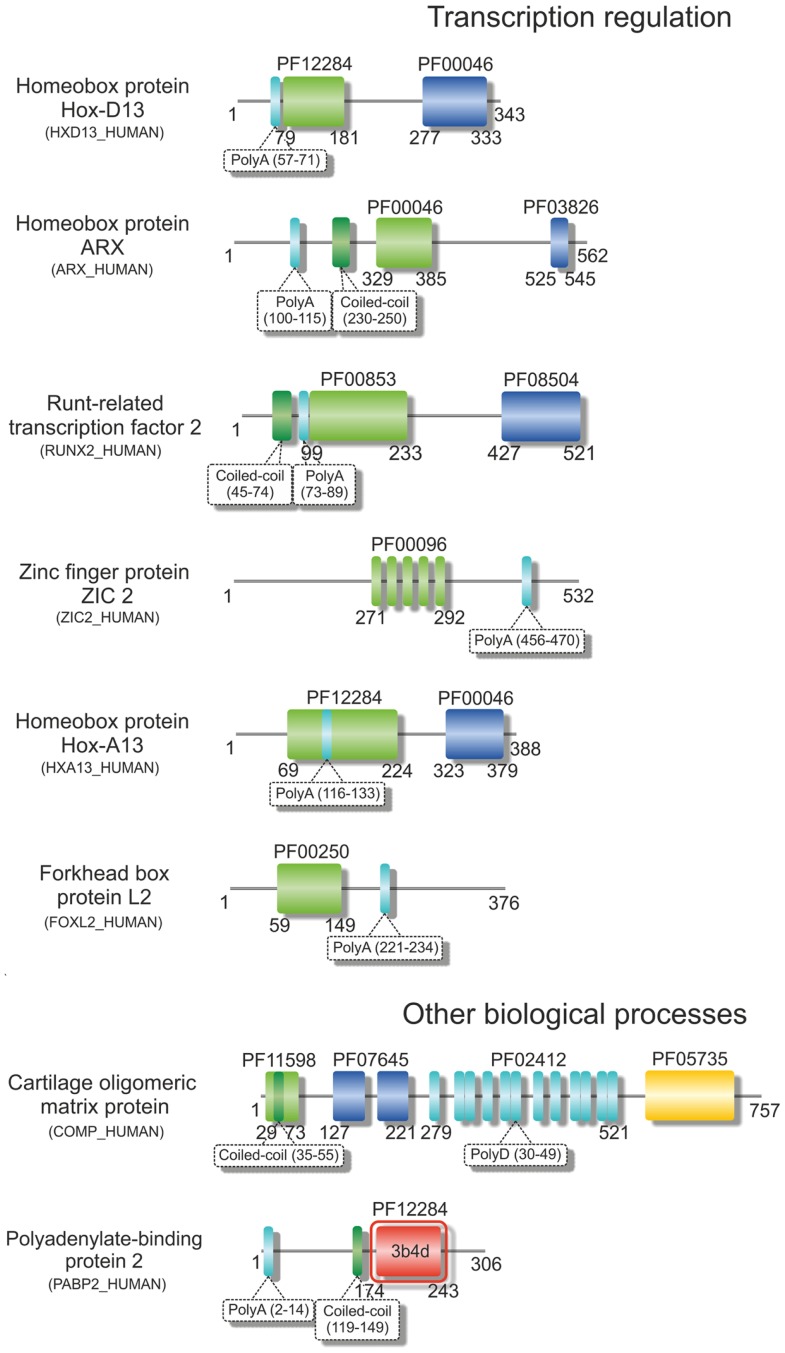
**Structural variability of proteins encoded by TNR-containing genes**. Illustrative domain graphics of the multi-domain structure of proteins associated with polyD- and polyA-expansion diseases. All proteins shown are referenced by their name as annotated in UniProt. The protein domains for which information is annotated in the Pfam database are shown as colored boxes with Pfam family accession code referenced above the domain box. Complete names of domains can be assessed by searching the specific Pfam accession code at http://pfam.sanger.ac.uk/. Numbers below the domain schemes represent amino acid residue numbers. Regions containing the amino acid repeats and with a prediction for formation of coiled-coils (as annotated in UniProt) are shown as well as regions with known 3D structure (boxed in red, with PDB accession codes shown). Notice the predominant location of the repeat regions within the N-terminal regions of the proteins.

Trinucleotide repeat (TNR) expansions and their association with neurological disorders have been known for the past 20 years (La Spada et al., 1991). Expansion of CAG, GCG, CTG, CGG, and GAA repeats located in coding or non-coding sequences of different genes (summarized in Table 1; Figures 1 and 2) are associated with a diverse range of human monogenic diseases such as Spinobulbar Muscular Atrophy (SBMA, a.k.a. Kennedy disease), Huntington Disease (HD), Spinocerebellar Ataxias (SCAs), Oculopharyngeal Muscular Dystrophy (OPMD), Myotonic Type 1 (DM1), Fragile X-Associated Tremor Ataxia Syndrome (FXTAS), and Friedreich Ataxia (FRDA) (for a review see Orr and Zoghbi, 2007), with longer repeats being correlated with earlier age at onset and increased disease severity. These TNR are highly unstable and the repeat tract length can change between affected individuals within the same family and can be different in different tissues (La Spada, 1997; Brouwer et al., 2009). More interestingly, in the brain of patients affected by CAG expansions, differences in repeat instability have been found between specific cell types (Pearson et al., 2005; Gonitel et al., 2008; Lopez Castel et al., 2010). GCG repeats are usually shorter and reveal a higher stability in different tissues and across generations than CAG repeats. The dynamic nature of these DNA repeat expansions is a consequence of their capability to form different secondary structures, which interfere with the cellular mechanisms of replication, repair, recombination and transcription (for a recent review see Lopez Castel et al., 2010). The molecular mechanisms underlying pathogenesis in those disorders, either associated with mental retardation, neuronal, or muscular degeneration, might result from alterations in the levels of gene expression and/or the function of the RNA or protein it encodes, mechanisms that likely act in concert to influence the pattern of selective cell toxicity. Some of those toxicity mechanisms will be briefly discussed below.

**Table 1:** **Human diseases associated with nucleotide repeat expansions (adapted from Messaed and Rouleau, 2009; Lopez Castel et al., 2010; Matos et al., 2011)**.

Disease name	Repeat type	Repeat location	Gene	Protein (UniProt identifier, number of residues)	Biological process^a^	Normal repeat length	Disease repeat length	Protein structure determined?
Spinal and bulbar muscular atrophy (SBMA)	CAG	Protein coding region (polyQ)	*AR*	Androgen receptor (P10275, 919 residues)	Transcription, transcription regulation	9–36	38–62	Residues 20–30 and 671–919 (PDB code 1xow)
Huntington’s disease (HD)	CAG	Protein coding region (polyQ)	*HTT*	Huntingtin (P42858, 3142 residues)	Apoptosis	6–34	36–121	Residues 5–18 (3lrh), Residues 1–17 (2ld0, 2ld2), Residues 1–64 (3io4, 3io6, 3ior, 3iot, 3iou, 3iov, 3iow)
Dentatorubral- pallidouysian atrophy (DRPLA)	CAG	Protein coding region (polyQ)	*ATN1*	atrophin 1 (P54259, 1190 residues)	Transcription, transcription regulation	7–34	49–88	No structural information
Spinocerebellar ataxia 1 (SCA1)	CAG	Protein coding region (polyQ)	*ATXN1*	ataxin 1 (P54253, 815 residues)	Transcription, transcription regulation	6–39	40–82	Residues 563–693 (1oa8)
Spinocerebellar ataxia 2 (SCA2)	CAG	Protein coding region (polyQ)	*ATXN2*	ataxin 2 (Q99700, 1313 residues)	No associated GO keywords for biological process	15–24	32–200	Residues 912–928 (3ktr)
Spinocerebellar ataxia 3 (SCA3)	CAG	Protein coding region (polyQ)	*ATXN3/MJD*	ataxin 3 (P54252, 364 residues)	Transcription, transcription regulation, Ubl conjugation pathway	10–51	55–87	Residues 1–182 (1yzb), Residues 222–263 (2klz)
Spinocerebellar ataxia 6 (SCA6)	CAG	Protein coding region (polyQ)	*CACNA1 A*	CACNA 1_A_, P/Q-type α1A calcium channel subunit (O00555, 2505 residues)	Calcium transport, ion transport, transport	4–20	20–29	Residues 1955–1975 (3bxk)
Spinocerebellar ataxia 7 (SCA7)	CAG	Protein coding region (polyQ)	*ATXN7*	ataxin 7 (O15265, 892 residues)	Transcription, transcription regulation	4–35	37–306	Residues 330–401 (2kkr)
Spinocerebellar ataxia 17 (SCA17)	CAG	Protein coding region (polyQ)	*ATXN17*	TATA box binding protein (TBP) (P20226, 339 residues)	Transcription, transcription regulation, Host-virus interaction	25–42	47–63	Residues 159–337 (1cdw, 1c9b, 1jfi, 1nvp, 1tgh)
Multiple skeletal dysplasias (COMP)	GAC	Protein coding region (polyaspartate)	*COMP*	cartilage oligomeric matrix protein (a.k.a Thrombospondin-5) (P49747, 757 residues)	Apoptosis, cell adhesion	5	4, 6, 7	Residues 225–757 (3fby).
Synpolydactyly (HOXD13)	GCG	Protein coding region (polyA)	*HOXD13*	homeobox D13 (P35453, 343 residues)	Transcription, transcription regulation	15	22–29	No structural information
Oculopharyngeal Muscular Dystrophy (OPMD)	GCG	Protein coding region (polyA)	*PABPN1*	Polyadenylate-binding protein 2 (Q86U42, 306 residues)	mRNA processing	10	12–17	Residues 167–254 (3b4d, 3b4m, 3ucg)
Cleidocranial dysplasia (CBFA1)	GCG	Protein coding region (polyA)	*RUNX2*	Runt-related transcription factor 2 (Q13950, 521 residues)	Transcription; transcription regulation	17	27	No structural information
Holoprosencephaly (ZIC2)	GCG	Protein coding region (polyA)	*ZIC2*	Zinc-finger protein ZIC 2 (O95409, 532 residues)	Differentiation, neurogenesis, transcription, transcription regulation	15	25	No structural information
Hand-Foot-Genital Syndrome/HOXA13)	GCG	Protein coding region (polyA)	*HOXA13*	homeobox A13 (P31271, 388 residues)	Transcription, transcription regulation	18	24–26	No structural information
Blepharophimosis/ptosis/epicanthus inversus syndrome type II (FOXL2)	GCG	Protein coding region (polyA)	*FOXL2*	Forkhead box like 2 (P58012, 376 residues)	Differentiation, transcription, transcription regulation	14	22–24	Residues 322–328 (2l7z)
Infantile spasm syndrome (ARX)	GCG	Protein coding region (polyA)	*ARX*	Aristaless-related homeobox (Q96QS3, 562 residues)	Differentiation, neurogenesis, transcription, transcription regulation	10–16	17–23	No structural information
Myotonic dystrophy type 1 (DM1)	CTG	3′UTR	*DMPK*	Myotonic dystrophy protein kinase (DMPK) (Q09013, 639 residues)	No associated GO keywords for biological process	5–37	90–6500	Residues 11–420 (2vd5), Residues 460–537 (1wt6)
Friedreich ataxia (FRDA)	GAA	Intron	*FXN*	Frataxin (Q16595, 210 residues)	Heme biosynthesis, Ion transport, Iron storage, Iron, transport	6–32	>200	Residues 88–210 (1ekg), Residues 91–210 (1ly7), Residues 82–210 (3s4m, 3s5d, 3s5e, 3s5f, 3t3j, 3t3k, 3t3l, 3t3t, 3t3x)
Spinocerebellar ataxia 8 (SCA8)	CTG	3′UTR	*ATXN8*	Ataxin-8 (a.k.a protein 1C2; (Present in SCA8-specific 1C2-positive intranuclear inclusions) (Q156A1, 80 residues)	Cell death	2–130	>110	Nostructural information
Spinocerebellar ataxia 12 (SCA12)	CAG	5′UTR	*PPP2R2B*	Serine/threonine-protein phosphatase 2A 55 kDa regulatory subunit B β isoform (Q00005, 443 residues)	Apoptosis	7–45	55–78	No structural information
Huntington disease-like 2 (HDL2)	CAG	Alternative splice isoform 2 – polyA-expansion	*JPH3*	Junctophilin 3 (Q8WXH2, 748 residues)	No associated GO keywords for biological process	6–27	51–57	No structural information
FRAXA: fragile X syndrome	CGG	5′UTR	*FMR1*	Fragile X mental retardation 1 protein (Q06787, 632 residues).	Transport; mRNA transport	6–52	230–2000	Residues 1–134 (2bkd), Residues 216–280 (2fmr), Residues 216–425 (2qnd), Residues 527–541 (2la5)
FXTAS: fragile X tremor/ataxia syndrome	CGG	5′UTR	*FMR1*	Fragile X mental retardation 1 protein (Q06787, 632 residues).	Transport; mRNA transport	6–52	59–230	Residues 1–134 (2bkd), Residues 216–280 (2fmr), Residues 216–425 (2qnd), Residues 527–541 (2la5)
FRAXE: fragile X syndrome	CGG	5′UTR	*FMR2*	Fragile X mental retardation 2 protein (P51816, 1311 residues)	mRNA processing, mRNA splicing	4–39	200–900	No structural information

*UTR, untranslated region*.

*^a^Biological Function based on Gene Ontology as annotated in UniProt*.

### Trinucleotide repeats and RNA structure

The formation of hairpin structures within the TNR RNA is related to the gain in RNA toxic function, the major pathogenic mechanism associated with CUG and CGG repeat expansions in non-coding regions of DM1 and FXTAS transcripts, which was also shown to contribute to pathogenesis in CAG repeat disorders such as HD and Machado-Joseph disease (MJD, a.k.a. SCA3) (reviewed in Krzyzosiak et al., 2012). These duplex structures, whose stability is positively correlated with the repeat size (Napierala and Krzyzosiak, 1997), sequester dsRNA binding proteins involved in mRNA splicing such as CUG-binding protein (CUGBP) and muscleblind protein 1 (MBNL1) (Miller et al., 2000), inducing aberrant splicing in affected cells, compromising multiple intracellular pathways, affecting cell-quality control regulation, and ultimately resulting in cell dysfunction (Li and Bonini, 2010). Structural studies on model trinucleotide CUG, CAG, and CGG repeats forming double-stranded chains revealed the features induced by periodic U-U, A-A, and G-G mismatches, and provided hints into the structural details of pathogenic RNAs that are recognized by RNA-binding proteins (Mooers et al., 2005; Kiliszek et al., 2010, 2011; Kumar et al., 2011; Parkesh et al., 2011). MBNL1 is composed of four zinc-containing RNA-binding domains arranged in two tandem segments, with the C-terminal zinc-finger pair displaying a GC-sequence recognition motif (Teplova and Patel, 2008) and interacting with the stem region of expanded CUG RNAs (Yuan et al., 2007). Electron microscopy analysis of MBNL1:CUG^136^ complexes showed that the pathogenic dsRNA forms a scaffold with tandem spaced MBNL1 binding sites were MBNL1 oligomers with a ring-like structure can assemble, possibly leading to the formation of the ribonuclear foci identified in cell models of these TNR diseases (Yuan et al., 2007; de Mezer et al., 2011). The structure and stability of the TNR hairpin structures formed depends on the presence of interruptions as well as on the nature of the flanking regions. This might be related with the ability of individual repeats to participate in the RNA toxicity mechanisms (Krzyzosiak et al., 2012).

In FRDA and FXTAS, pathogenesis results predominantly from decreased expression of the associated genes (*FXN* and *FMR1/FMR2*) caused by the expansion of GAA and CGG repeats, respectively, which results in loss of function of key proteins involved in iron-sulfur cluster biogenesis and mRNA translation at synapses. Nevertheless, in FXTAS RNA toxicity is also proposed to play a role in pathogenesis (Li and Bonini, 2010). The recently discovered mechanisms of pathogenesis in spinocerebellar ataxia type 8 (SCA8) uncovered the extreme complexity of TNR disorders. In fact, SCA8 is caused by expansion of CTG/CAG repeats in the affected gene, which are transcribed bi-directionally leading to the generation of expanded CUG and CAG-containing transcripts further translated into homopolymeric proteins, so that pathogenesis can be mediated by both RNA and protein toxicity (Merienne and Trottier, 2009). Curiously, recent data have highlighted the possibility of non-ATG translation across expanded TNR in all possible reading frames, which might further contribute to the generation of novel toxic proteins and RNAs adding to the multi-parametric character of the pathogenic mechanisms associated with TNR diseases (Li and Bonini, 2010; Pearson, 2011; Sicot et al., 2011).

### Trinucleotide repeats within protein coding regions

Over 20 years ago, the finding that the expansion of CAG repeats within the coding sequence of the androgen receptor gene was the genetic basis of SBMA (La Spada et al., 1991) represented a hallmark in the discovery of these novel dynamic mutations and their association with human disease. Some years later, the identification of intracellular inclusions containing the expanded proteins (Paulson et al., 1997) provided a clue to pathogenesis, directing research in the field into an extensive search for the mechanisms of polyQ-induced protein aggregation. The moderate expansion of GCG and CAG repeats, which are translated into polyA and polyQ tracts in the affected proteins (Figures 1 and 2), results in protein misfolding and aggregation, in accordance with a general, although not always unique, toxic gain of function mechanism of pathogenesis (Williams and Paulson, 2008). The appearance of insoluble cytoplasmic or nuclear inclusions enriched in the expanded polyA- or polyQ-containing protein constitutes a characteristic fingerprint of these diseases (Messaed and Rouleau, 2009; Orr, 2012a), regardless of their controversial role in pathogenesis. While the proteins containing polyA repeats are predominantly transcription factors with a role in development (see Table 1 and Amiel et al., 2004; Messaed and Rouleau, 2009), most of the proteins linked to polyQ-expansion diseases are involved in DNA-dependent regulation of transcription or neurogenesis and often contain multiple intermolecular partners (Butland et al., 2007). Despite the overall lack of sequence or structural homology, both polyQ- and polyA-repeat expansions are associated with formation of ß-rich amyloid-like protein inclusions, and with the wider group of protein misfolding disorders. These inclusions are enriched in ubiquitin, proteasome subunits, and chaperones, and often recruit macromolecules that are part of the macromolecular interaction networks associated with the proteins’ native functions (Williams and Paulson, 2008). As an example, the poly(A)-binding protein PABNP1 forms insoluble inclusions upon alanine expansion, co-aggregating together with poly(A)-mRNA, proteasome subunits, ubiquitin, heat-shock proteins, and SKIP, a transcription factor associated with muscle-specific gene expression (Brais, 2003; Tavanez et al., 2009; Winter et al., 2013).

The simplistic view of the predominant role of the inclusions in polyQ-induced pathogenesis was later challenged by the failure of this mechanism to explain the cell-specific vulnerability characteristic for each disease and by the identification of numerous examples of neuronal toxicity in the absence of visible intracellular inclusions (Arrasate et al., 2004). Indeed, the inclusions were shown to be fibrillar and display amyloid-like properties both *in vivo* and *in vitro* (Huang et al., 1998; Bevivino and Loll, 2001; Sathasivam et al., 2010) and, in a mechanistic parallel with the pathogenic mechanisms proposed for “classical” amyloids, many studies suggested that the insoluble inclusions played a protective role, sequestering toxic, and misfolded protein conformers (Arrasate et al., 2004; Rub et al., 2006; Miller et al., 2010). Indeed, soluble intermediates in the aggregation pathway such as misfolded β-sheet rich polyQ protein monomers and oligomers have latter been identified and proposed to represent the major toxic species (Kayed et al., 2003; Gales et al., 2005; Nagai et al., 2007; Miller et al., 2011). Also, in OPMD, the primary toxic species are proposed to be the soluble variants of the expanded polyA-repeat protein PABPN1 (Messaed et al., 2007). It is currently accepted that in polyQ disorders the expanded region plays a role in inducing the self-assembly of the carrier protein, which engages in pathogenic interactions and leads to the formation of toxic monomers or oligomers (Takahashi et al., 2008; Weiss et al., 2008) latter converted to insoluble intracellular amyloid-like oligomers where both expanded and “normal” protein are sequestered along with other macromolecular partners (reviewed in Williams and Paulson, 2008; Matos et al., 2011; Costa and Paulson, 2012). As more biochemical data is gathered, more is understood about the role of amino acid expansions in modulating the interaction with macromolecular partners. As an example, expansion of the polyA tract in PABPN1 results in increased association with Hsp70 chaperones and type I arginine methyl transferases (Tavanez et al., 2009). This indicates that the distinct neuropathological features arising from this amino acid-repeat expansion might at least partially result from alterations on the native biological functions and macromolecular interactions of the carrier protein, which might vary in different intracellular environments.

Recent data have shown that expansion of polyA repeats is frequently associated with loss of normal function altering a multitude of cellular pathways with consequences in cell functionality (Amiel et al., 2004; Messaed and Rouleau, 2009), although protein aggregation might also play a dominant role in some of the polyA-associated disorders (Messaed and Rouleau, 2009; Winter et al., 2013). Studies with polyQ proteins have shown that pathogenesis might result from a subtle imbalance in the association of the mutant protein with multiple cellular partners and that toxicity and neuronal death could result from a combination of protein self-assembly and functional alterations (Friedman et al., 2007; Li et al., 2007b; Lim et al., 2008; Kratter and Finkbeiner, 2010; Orr, 2012b; Pastore and Temussi, 2012). In fact, neuronal death as a result of polyQ-expansion seems to resemble that of linker cell in *C. elegans* (Pilar and Landmesser, 1976; Chu-Wang and Oppenheim, 1978; Blum et al., 2012, 2013) which involves the polyQ protein pqn-4, pointing for a common mechanism for linker cell death, and neuronal death in polyQ diseases (Blum et al., 2013).

Polyglutamine diseases constitute a representative and largely studied group of neurodegenerative disorders where considerable amounts of data have been collected on the role of expanded polyQ for disease pathogenesis. However, given the proposed function of polyQ regions in mediating protein–protein interactions, which might be modulated by polyQ-expansion (Schaefer et al., 2012), the information on the role of these regions for native protein function, structure, and dynamics is still limited. Structural and functional information on the role of these repeat sequences in protein function is crucial to better understand how expansion affects selected neuronal subpopulations. Below, we briefly discuss the current knowledge on the function and structure of polyQ repeats and their role on macromolecular interactions, and finally focus on the known structural and functional information on ataxin-3, the protein whose mutation causes MJD.

## Function of PolyQ on Protein–Protein Interactions and Evolution

Until recently, the function of many amino acid-repeat-containing proteins and the role of homopeptide regions were somewhat obscure. However, several global analysis studies on single amino acid-repeat-containing proteins shed light onto their function and onto the biological significance of the repeated region, in particular of polyQ, the most prevalent amino acid repetition in humans (Alba and Guigo, 2004). It is now accepted that TNR, particularly those located within protein-coding regions, are considered important mutators providing the genetic variability required for driving evolution (King, 1994; Kashi et al., 1997; Kashi and King, 2006; Nithianantharajah and Hannan, 2007). In fact, simple or low-complexity amino acid-repeats are rare within prokaryotic but extremely abundant within eukaryotic proteins, particularly over-represented in *Plasmodium* (49–90% of the total proteome), *D. discoideum* (52%), *D. melanogaster* (20%), *C. elegans* (9%), and *H. sapiens* (14%) (Haerty and Golding, 2010). Among all homopolymeric repeats, the most common on eukaryotic proteins are glutamine, asparagine, alanine, and glutamate repeats (Faux et al., 2005). This seems to indicate that there has been a strong negative selection against the appearance of hydrophobic amino acid-repeats with high tendency to aggregate, such as polyisoleucine, polyleucine, polyphenylalanine, and polyvaline (Oma et al., 2005, 2007).

The homopeptide regions seem to be particularly relevant for brain development and function, since these repeated regions can be found in various neurodevelopmental genes (Nithianantharajah and Hannan, 2007). Indeed, the sexual behavior of prairie voles (Hammock and Young, 2005), as well as human pair-bonding (Walum et al., 2008), seems to be dependent on the repeat length in the vasopressin 1A receptor gene. A wide study of the distribution and function of homopeptide-containing proteins could also demonstrate a clear trend in humans, *D. melanogater*, and *C. elegans*, with the majority of homopeptide-containing proteins performing roles in transcription/translation and signaling processes and to a less extend in transport and adhesion processes (Faux et al., 2005). A similar profile was also found in a comparative analysis of proteins with amino acid-repeats in human and rodents (Alba and Guigo, 2004) and also on a comparative genomic study in domestic dogs, which unveiled an association between morphological variations and the length of the repeated region in the transcription factor-encoded genes *ALX4* and *RUNX2* (Fondon and Garner, 2004). Analysis of the human genome also revealed the existence of 64 CAG repeat-containing genes involved in biological processes such as regulation of transcription, binding of transcriptional co-activators and transcription factors, and in neurogenesis in general (Butland et al., 2007). Additionally, a detailed analysis of the human polyQ database (http://pxgrid.med.monash.edu.au/polyq/) (Robertson et al., 2011) also indicated that the majority of polyQ-containing proteins display domains involved in development (Homeobox domain-containing proteins, Fibroblast growth factor receptor), chromatin remodeling (Bromodomain and PHD-containing proteins), and signal transduction (PDZ domain-containing proteins), all biological processes that are highly dependent on protein–protein interactions and associated with the formation of multicomponent protein complexes. As for humans, analysis of bovine polyQ proteins revealed an enrichment for large multi-domain transcriptional regulators (Whan et al., 2010).

It is currently accepted that the majority of repeat-containing proteins perform roles in processes that require the assembly of large multiprotein or protein/nucleic acid complexes (Faux et al., 2005; Hancock and Simon, 2005; Whan et al., 2010). Supporting this notion is the fact that homopolymeric amino acid-repeats are considered to be unstructured (Gojobori and Ueda, 2011) and that intrinsically unstructured regions are suggested to constitute macromolecular docking sites, which become structured only when bound to cognate ligand partners (Huntley and Golding, 2002; Simon and Hancock, 2009). In fact, “hub proteins” contain significantly longer and more frequent repeats or disordered regions, which facilitate binding to multiple partners (Dosztanyi et al., 2006). Recently, Fiumara et al. (2010) found an overrepresentation of coiled-coils domains in polyQ-containing proteins and in their interaction partners, which are able to form α-helical supersecondary structures, often inducing protein oligomerization (Parry et al., 2008). Thus, polyQ tracts due to their intrinsic structural flexibility, which is largely influenced by the flanking residues (see PolyQ: A Simple Sequence Repeat with a Polymorphic Structure below), may act as stabilizers of intra- and intermolecular protein interactions, possibly by extending a neighboring coiled-coil region to promote its interaction with a coiled-coil region in an interacting protein partner (Schaefer et al., 2012). A detailed analysis revealed heptad repeats typical of coiled-coils in regions flanking or overlapping polyQ stretches, whose disruption is sufficient to impair CHIP-huntingtin interaction, indicating that coiled-coils are crucial for polyQ-mediated protein contacts. Importantly, coiled-coils also seem to be important for the regulation of aggregation and insolubility of polyQ-containing proteins (see below and Fiumara et al., 2010) as recently proposed by Petrakis et al. (2012), which discovered a recurrent presence of coiled-coil domains in ataxin-1 misfolding enhancers, while such domains were not present in suppressors.

Based on the several observations on the function of polyQ-containing proteins it is suggested that a general function of polyQ, as for the majority of repeat sequences, is to aid in the assembly of macromolecular complexes, either through tethered distant domains or through interactions with the polyQ itself (Gerber et al., 1994; Korschen et al., 1999; Faux et al., 2005). By affecting protein interactions, and being present in particular functional classes such as transcription factors, polyQ is considered central to the evolution of this type of proteins and consequently crucial to the evolution of cellular signaling pathways (Hancock and Simon, 2005).

A structural analysis of polyQ repeats and its flanking domains as well as its role in protein aggregation will be discussed in greater detail in the next sections.

## Structural Studies on PolyQ Repeats

Since the discovery that polyQ repeats are associated with human neurodegenerative diseases that a huge effort has been made to determine the structure of polyQ and to understand how expansion of the repeat affects the structure of the carrier protein and/or the normal interaction with molecular partners. The first evidence from the aggregation-prone character of polyQ-rich proteins came from studies with glutamine-rich cereal storage proteins and synthetic glutamine polypeptides (Beckwith et al., 1965; Krull et al., 1965). After the discovery that a number of neurological disorders were triggered by expansion of a polyQ tract in different and unrelated proteins (La Spada et al., 1994), and before intracellular inclusions enriched in the polyQ-expanded protein were identified as a major fingerprint in these diseases (Davies et al., 1997; Paulson et al., 1997), Perutz (1994) anticipated that the expanded polyQ tract could mediate protein–protein interactions causing protein aggregation in neurons and recruiting other polyQ-rich proteins such as transcription factors leading to cellular dysfunction. Below, the structural features and self-assembly properties of polyQ sequences are briefly discussed (for a detailed review on the biophysical and structural features of polyQ, see Wetzel, 2012).

### PolyQ: A simple sequence repeat with a polymorphic structure

In order to elucidate the structure of the glutamine repeat and to uncover the structural changes induced by polyQ expansion, several strategies have been put forward including (a) the structural analysis of polyQ-containing peptides of different lengths, (b) the characterization of proteins of well-known structure after insertion of an exogenous polyQ repeat, and structural determination of (c) polyQ-antibody complexes, or (d) natural polyQ-rich proteins.

Using synthetic peptides containing 15 glutamine repeats, Perutz and coworkers proposed that polyQ stretches could self-associate forming hydrogen bonds between their side-chain amide groups and the main chain of a neighboring β-strand, to form cross-β structures (polar zippers) (Perutz, 1994). This study was followed by many reports where synthetic polyQ peptides were used as models of the biophysical properties of polyQ-rich proteins, which established that polyQ-containing peptides have a tendency toward self-assembly into amyloid-like structures (Chen et al., 2002a). Moreover, the results obtained *in vitro* reflected disease features observed *in vivo* such as the correlation between larger polyQ size, increased protein aggregation, and earlier disease onset (Chen et al., 2002b; Kar et al., 2011). Circular dichroism studies of polyQ peptides in solution have shown that their monomeric forms lack regular secondary structure (Altschuler et al., 1997; Klein et al., 2007) and additional biophysical experiments proposed that these peptides can adopt collapsed (Crick et al., 2006; Dougan et al., 2009; Peters-Libeu et al., 2012) or extended (Singh and Lapidus, 2008) coils in solution whose compactness was strongly correlated with the polyQ size (Walters and Murphy, 2009). The determination of the structure of monomeric polyQ peptides with atomic detail is however still lacking as a result of their intrinsic conformational flexibility and tendency to aggregate into heterogeneously sized β-rich oligomers. From the combination of experimental and theoretical methods a picture for polyQ structure and aggregation is emerging, where the monomeric polyQ adopt an ensemble of conformations lacking regular secondary structures that assemble into β-structures in a polyQ-length dependent fashion (Vitalis et al., 2009; Walters and Murphy, 2009, 2011; Williamson et al., 2010; Kar et al., 2011). Divergent results proposing the existence of predominantly extended or collapsed conformations or the minimum size for polyQ aggregation are likely due to the differences in the introduction of variable flanking residues (Kar et al., 2011). They might result from the insertion of different polyQ tract interrupting residues (Walters and Murphy, 2011), or be a consequence of the protocols used for the preparation and disaggregation of the peptides used for the biophysical studies (Jayaraman et al., 2011). Most results obtained with these peptides do not generally take into account the possible effects of the protein context on the structural properties of the polyQ stretches, a particularly relevant feature considering that the role of non-polyQ domains in protein aggregation has been reported for ataxin-1 (de Chiara et al., 2005), ataxin-3 (Gales et al., 2005), and huntingtin (Tam et al., 2009; Thakur et al., 2009; Liebman and Meredith, 2010).

**Figure 3:**
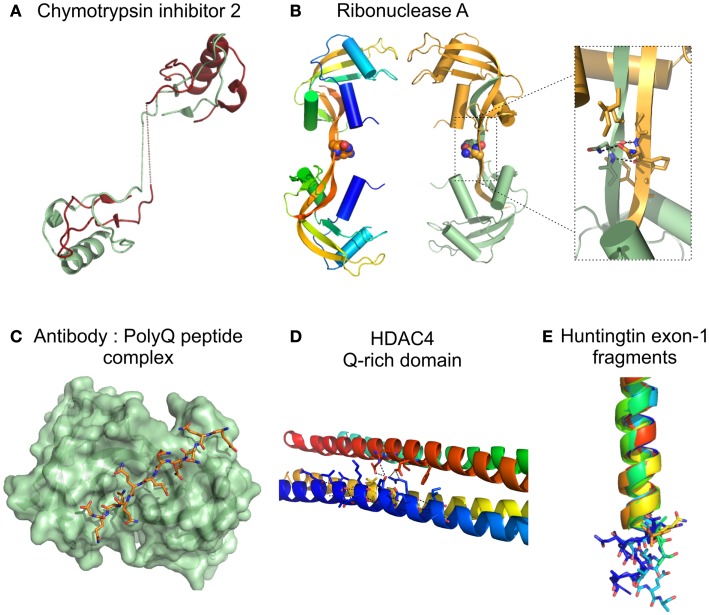
**Structure of proteins/protein domains containing polyQ regions**. **(A)** Cartoon representation of the domain swapped dimer of chymotrypsin inhibitor 2 with a 4 glutamine insertion [(Chen et al., 1999); PDB accession code 1cq4], dotted lines represent the polyQ linker not visible in the X-ray crystal structure. **(B)** Cartoon representation of domain swapped major dimers of ribonuclease A. Inset shows a short segment resembling the polar zipper formed by asparagine residues in the linker region [(Liu et al., 2001); PDB accession code 1f0v]. **(C)** Surface representation Fv fragment of a monoclonal antibody in complex with a polyQ peptide shown as sticks [(Li et al., 2007a), PDB accession code 2otu]. **(D)** Cartoon representation of the glutamine-rich domain from HDAC4 showing details of the polar interactions (dotted lines) at the oligomer interfaces involving glutamine residues [(Guo et al., 2007), PDB accession code 2o94]. **(E)** Cartoon representation of the crystal structures of huntingtin exon-1 fragments observed in different crystal forms, highlighting the different orientations of the C-terminal polyQ residues shown as sticks. The 17 glutamine stretch adopts variable conformations in the structures: α helix, random coil, and extended loop. [(Kim et al., 2009), PDB accession codes 3io4, 3iow, 3iov, 3iou, 3iot, 3ior, 3io6].

In a pioneer work, Stott et al. (1995) inserted a G-Q_10_-G peptide into the inhibitory loop of chymotrypsin inhibitor 2 (CI2), a soluble small protein from barley seeds, showing that this CI2-polyQ chimera has an increased tendency for self-assembly. Even though a CI2 variant with four glutamines crystallized, the structure of the CI2-Q_4_ dimer showed that the polyQ region was disordered and that oligomerization was mediated by domain swapping (Figure 3A) and not by direct polyQ association (Chen et al., 1999). A structure resembling the proposed polar zipper was later observed between two asparagines in the hinge loop of the major domain swapped dimer of bovine pancreatic ribonuclease A (Liu et al., 2001) (Figure 3B). Insertion of a 10 glutamine repeat within this hinge loop of ribonuclease A, resulted in domain swapping, oligomerization, and amyloid-like fiber formation, but strikingly the enzyme within the fibers was catalytically active, retaining its native fold (Sambashivan et al., 2005). However, although the structure of the domain swapped dimer was solved by X-ray crystallography, the repeat region was not visible in the electron density maps.

A first overview of a short polyQ stretch at atomic resolution resulted from the structure of a polyQ_10_ peptide (GQ_10_G) (Figure 3C) bound to MW1, an antibody against polyQ. This structure reveals that polyQ adopts an extended, coil-like structure in which contacts are made between side chains and/or main chain atoms of all 10 glutamines and the antibody-combining site (Li et al., 2007a). The peculiar structural features of these repeat-containing regions were also revealed by the crystallographic structure of a glutamine-rich domain of human histone deacetylase (HDAC4), that folds into a tetramer-forming straight α-helix (Figure 3D). The protein interfaces consist of multiple hydrophobic patches separated by polar interaction networks, in which clusters of glutamines engage in extensive intra- and interhelical interactions (Guo et al., 2007). Further details on the structure of polyQ were unveiled by the high-resolution crystal structures of huntingtin (HD) exon 1, containing 17 glutamines (Htt17Q) (Kim et al., 2009). Htt17Q in fusion with maltose-binding protein (MBP) folds into an amino-terminal α-helix followed by a polyQ_17_ region that adopts multiple conformations in the different crystal forms, including α-helix, random coil, and extended loop, and a polyproline helix formed by the polyP_11_ and mixed P/Q regions (Figure 3E). The authors suggested that the shallow equilibrium between α-helical, random coil, and extended conformations can be subtly altered by the size of polyQ sequence, the neighboring protein context, protein interactions, or by changes in cellular environment, and that this polymorphic behavior is a common characteristic of many amyloidogenic proteins (Kim et al., 2009).

### Self-assembly and aggregation of polyQ repeats

The first approaches to characterize polyQ-induced protein aggregation and pathogenesis in the context of a full-length protein included the insertion of the polyQ peptides into well-known non-pathogenic protein carriers such as hypoxanthinephosphoribosyl transferase (HPRT), which resulted in a neurological phenotype mimicking that observed in mice expressing the mutant HD truncated protein (Ordway et al., 1997). *In vitro* studies aiming at better characterizing the structure and function of polyQ repeats in the context of full-length soluble proteins, included the insertion of ectopic polyQ stretches into well-characterized and soluble proteins such as CI2 (Stott et al., 1995; Chen et al., 1999), myoglobin (Mb) (Tanaka et al., 2001; Tobelmann and Murphy, 2011), glutathione S transferase (GST) (Masino et al., 2002; Bulone et al., 2006) and the B domain from *Staphylococcus aureus* Protein A (SpA) (Saunders et al., 2011). Fusion of the polyQ sequences with stable and soluble proteins moderates the intrinsic polyQ peptide aggregation propensity, but induces the self-assembly of carrier proteins into fibrillar amyloid-like structures, a nucleation-dependent process whose kinetics is directly proportional to the size of the inserted polyQ repeat. Likewise, polyQ peptides are able to seed the aggregation of intracellular soluble polyQ-containing proteins when added to cell cultures, conferring a heritable phenotype of self-sustaining seeding, resembling a prion-like mechanism (Ren et al., 2009), reviewed in Cushman et al. (2010).

The impact of the polyQ tract and its expansion on the perturbation of the structure of flanking sequences and domains is critically dependent on the location of the amino acid-repeats, revealing impressive location-dependent changes in structural stability, and fibril morphology of the host proteins (Robertson et al., 2008; Saunders et al., 2011; Tobelmann and Murphy, 2011). Curiously, the studies with these model proteins showed that stability and structure of the carrier protein remained unaltered by polyQ expansion when the repeat was inserted at the N- or C-terminus of the structured domain (Robertson et al., 2008), mimicking the location of polyQ tracts in most disease-related proteins (Figure 1).

The role of the flanking regions in modulating protein fibril formation in polyQ disease proteins is well supported by experimental data (de Chiara et al., 2005; Gales et al., 2005; Bhattacharyya et al., 2006; Saunders and Bottomley, 2009; Tam et al., 2009; Thakur et al., 2009; Liebman and Meredith, 2010), in agreement with the knowledge that different polyQ-containing proteins have a diverse threshold for aggregation. For example, addition of a polyproline extension after the polyQ repeat slows down aggregation (Bhattacharyya et al., 2006), while protein domains outside the polyQ tract [e.g., Josephin domain (JD) of ataxin-3 and AHX domain of ataxin-1] have been shown to contribute to protein aggregation (Masino et al., 2004; de Chiara et al., 2005; Gales et al., 2005; Ellisdon et al., 2006, 2007). The multitude of data on the polyQ-induced aggregation of disease and non-disease-proteins highlights the complex interplay between the polyQ region and the adjacent protein domains. In light of the polymorphic nature of the polyQ and the modulation of its structural features by the protein context, two general mechanisms have been proposed for polyQ-mediated toxicity (Kim et al., 2009): (a) the expanded polyQ stretch adopts a novel conformation that mediates toxicity or is the precursor to toxic species; (b) intra- or intermolecular protein interactions mediated by expanded polyQ in the random coil conformation are sufficient to result in pathological effects. In both cases the affinity of the interactions involving the expanded polyQ region could be higher with selected target proteins, leading to a preference of the disease proteins for some of the protein partners, a fact that is in agreement with the hypothesis raised by Zuchner and Brundin (2008), which postulate that resistance to NMDA receptor-mediated excitotoxicity occurring in some mouse models for HD is a consequence of a differential binding of partner proteins, in a polyQ tract size dependent manner, to the proline-rich domain of huntingtin. In this context, differences in molecular interactions occurring in a cell- and tissue-specific manner would result in different toxicities according to particular cellular environments.

Given the above mentioned studies, it is nowadays clear that the polyQ region influences aggregation of proteins, but this process is highly dependent on the surrounding protein context. Therefore, even though the structural information on peptides and proteins with polyQ expansions is a useful guideline for the investigation of the pathogenic effects of polyQ expansion, each of the proteins involved in polyQ diseases shows distinctive characteristics, cellular roles, and structural properties causing difficulties in the formulation of structural hypothesis that could explain how different monomeric conformations of polyQ leads to various aggregated species and how they contribute to neurotoxicity.

## PolyQ Repeats in Ataxin-3 Function and Dysfunction

Machado-Joseph disease is an inherited neurodegenerative disorder of adult onset originally described in people of Portuguese Azorean descent but later shown to be the most common autosomal dominant spinocerebellar ataxia worldwide. Clinically, it is characterized by ataxia, ophthalmoplegia, and pyramidal signs, associated in variable degree with dystonia, spasticity, peripheral neuropathy, and amyotrophy (Coutinho and Andrade, 1978). Pathologically, the disorder is associated with degeneration of the deep nuclei of the cerebellum, pontine nuclei, subthalamic nuclei, substantia nigra, and spinocerebellar nuclei (Coutinho et al., 1982; Rosenberg, 1992; Margolis and Ross, 2001). It is caused by an expansion of a repetitive CAG tract within the *ATXN3* gene (Kawaguchi et al., 1994). While in the healthy population the number of CAG repeats ranges between 10 and 51, in MJD patients the length of ataxin-3 polyQ tract exceeds 55 consecutive residues. Ataxin-3 is a modular protein, located both in the nucleus and the cytoplasm (Perez et al., 1999; Antony et al., 2009; Macedo-Ribeiro et al., 2009), encompassing an N-terminal globular JD, with structural similarity to cysteine proteases (Scheel et al., 2003; Albrecht et al., 2004), followed by an extended tail composed of two ubiquitin interaction motifs (UIMs), the expandable polyQ tract, and a C-terminal region (Matos et al., 2011). The C-terminal region of ataxin-3 may contain a third UIM, depending on the splice variant (Goto et al., 1997), with the 3UIM isoform of ataxin-3 being predominantly found in the brain (Harris et al., 2010). Currently, the physiological function of ataxin-3, as well as the molecular mechanism by which expanded polyQ sequences causes selective neurodegeneration remain mostly unknown. However, since it is ubiquitously expressed and cell death is region specific, neurodegeneration is currently viewed as depending on sequence and structural features outside the ataxin-3 polyQ tract [reviewed in Matos et al. (2011) and references therein].

### Ataxin-3 biological roles

**Figure 4:**
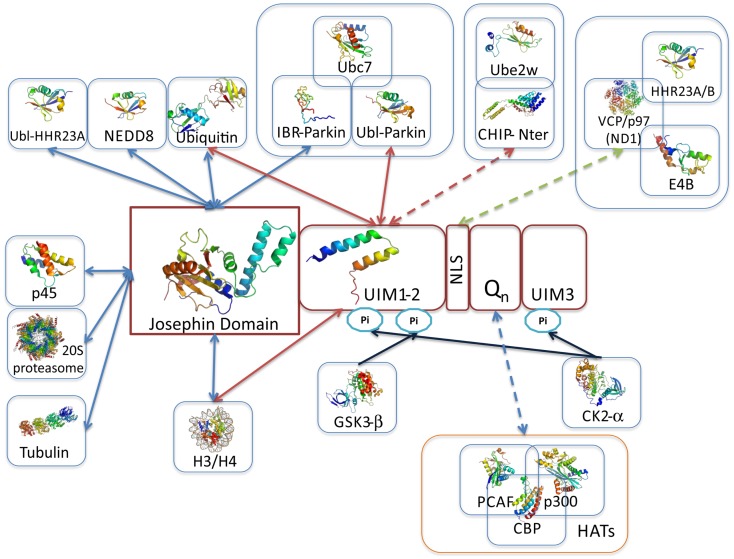
**Overview of ataxin-3 structural information**. Schematic illustration of ataxin-3 (isoform 2; a.k.a. 3UIM isoform) domain structure highlighting the regions involved in protein–protein interactions. The solution structures of the Josephin domain (PDB accession code 1yzb) and UIMs1-2 (PDB accession code 2klz) are shown colored from N-(blue) to C- terminus (red). JD-, UIM-, NLS-, and polyQ-mediated interactions are represented by blue, red, green, and purple arrows, respectively; blue arrows indicate the location of post-translational modification sites, resulting from the interaction and phosphorylation by CK2 and GSK3. Representative multi-subunit complexes where ataxin-3 participates are boxed (Li et al., 2002; Matsumoto et al., 2004; Scaglione et al., 2011; Durcan et al., 2012). One of the main questions in the quest for ataxin-3 interacting proteins is whether polyQ-expansion of the disease-protein modulates the binding affinities. Current data indicates that polyQ-expansion increments the ataxin-3 affinity for CHIP (Scaglione et al., 2011), VCP/p97 (Matsumoto et al., 2004; Boeddrich et al., 2006; Zhong and Pittman, 2006), and the transcription regulators p300, CBP, and PCAF (Li et al., 2002) (interactions represented by broken lines). Strikingly, all these interactions are mediated by ataxin-3 flexible tail, which includes the polyQ tract. Moreover the transcriptional regulators p300, CBP, and NCOR all contain amino acid repeats.

*ATXN3* orthologs have been identified in eukaryotic organisms including protozoans, plants, fungi, and animals (Albrecht et al., 2004; Costa et al., 2004; Rodrigues et al., 2007). Several functions have been ascertained to ataxin-3 based on studies with orthologs. Specifically, a role in cell structure and/or motility was proposed for mouse ataxin-3 as it is highly abundant in all types of muscle and in ciliated epithelial cells (Costa et al., 2004). In fact, ataxin-3 is able to interact with tubulin through its JD domain (Figure 4), with nM affinity (Mazzucchelli et al., 2009), which supports a role in cell structure. Interestingly, data on ataxin-3 *C. elegans* ortholog not only reinforces a function in structure/motility and signal transduction (Rodrigues et al., 2007), but also indicate a function in development as absence of *ATXN3* strongly modifies expression of several development-related genes. *ATXN3* knock-out animals showed no obvious deleterious phenotype, probably due to a putative redundant function between ataxin-3 and other JD-encoding proteins, such as ataxin-3-like protein, Josephin 1 and Josephin 2, all containing a typical cysteine protease catalytic triad. However the studies with *ATXN3* knock-out animals revealed an overall increase in the levels of ubiquitinated proteins (Schmitt et al., 2007) and signs of altered expression of core sets of genes associated with the ubiquitin-proteasome and signal transduction pathways (Rodrigues et al., 2007), pointing to a dual function of ataxin-3 in the ubiquitin-proteasome system and transcriptional regulation (Matos et al., 2011; Orr, 2012a).

#### Ataxin-3 function as transcriptional regulator

**Figure 5:**
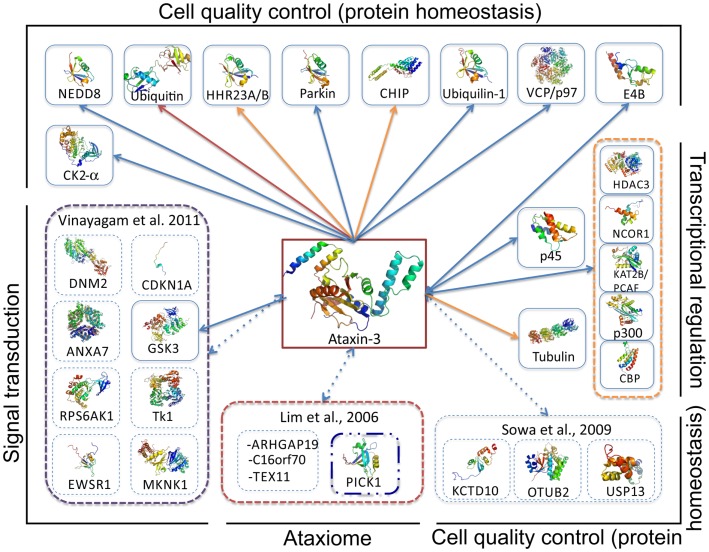
**Overview of ataxin-3 protein interaction network**. Data on the ataxin-3 interactors was obtained by analysis of Interactome3D (Mosca et al., 2012), MINT (Ceol et al., 2010), and Dr. PIAS (Sugaya and Furuya, 2011) protein interaction databases, and completed with data compiled from current literature on ataxin-3 protein associations obtained with a diverse set of experimental approaches (see complete information on Table 2). Red arrows indicate interactions for which structural data has been obtained, while orange arrows indicate that biophysical data on interaction affinity *in vitro* is known (Table 2). Broken arrows represent interactions that result from high-throughput interactome analysis that still require detailed biochemical and functional analysis. Proteins are grouped according to their biological role.

The putative role of ataxin-3 in transcriptional regulation is proposed to entail the modulation of histone acetylation and deacetylation at selected promoters. Ataxin-3 interacts with the major histone acetyltransferases cAMP-response-element binding protein (CREB)-binding protein (CBP), p300, and p300/CREB-binding protein-associated factor (KAT2B/PCAF, Figures 4 and 5), and is proposed to inhibit transcription in specific promoters (e.g., MMP-2 promoter) either by blocking access to histone acetylation sites or through recruitment of histone deacetylase 3 (HDAC3) and nuclear receptor co-repressor (NCOR1; Figures 4 and 5) (Li et al., 2002; Evert et al., 2006). Although, the interaction sites have not been mapped in detail for all these proteins, co-immunoprecipitation experiments showed that KAT2B/PCAF, p300, and CBP bind exclusively to the polyQ-containing C-terminal region of ataxin-3 (Figure 4), apparently in a polyQ-size dependent manner (Li et al., 2002). Experimental evidence also indicates that ataxin-3 forms part of a CREB-containing complex, although no direct interaction has been observed between the two proteins (Li et al., 2002). In contrast, the N-terminal region of ataxin-3 directly binds histones H3 and H4 (Table 2; Figure 4) (Li et al., 2002). Of note, p300 and CBP, as well as NCOR1, also encompass amino acid repetitions in its sequence. Interestingly, in huntingtin and in ataxin-1, polyQ interferes with CBP-activated gene transcription via interaction of their glutamine-rich domains (Shimohata et al., 2000; Nucifora et al., 2001) and mutant huntingtin targets specific components of the core transcriptional machinery, in a glutamine-tract length-sensitive manner (Zhai et al., 2005), pinpointing once again the role of the amino acid-repeat region in the establishment of protein–protein interactions.

**Table 2:** **Human ataxin-3 associated proteins**.

Ataxin-3 interacting protein (UniProt accession code)	Protein name	Direct interaction?	Interaction domains		Reference
			Ataxin-3	Partner protein	
**CELL-QUALITY CONTROL (PROTEIN HOMEOSTASIS)**					
HHR23A/B (P54725/P54727)	UV excision repair protein RAD23 homolog A/B	Yes, kD (JD:Ubl) = 12 μM	JD	Ubiquitin-like (Ubl) N-terminal domain	Wang et al. (2000), Doss-Pepe et al. (2003), Nicastro et al. (2005, 2009)
Poly-ubiquitin (P0CG48/P0CG47)	Polyubiquitin-C/Polyubiquitin-B	Yes, kD (atxn3:K48-tetraUb) = 0.2 μM, kD (atxn3:Ub) = 50 μM	UIMs, JD	K48- and K63-linked Ub (≥4 Ub), K48-linked diUb	Burnett et al. (2003), Doss-Pepe et al. (2003), Chai et al. (2004), Nicastro et al. (2009, 2010)
Ubiquilin-1 (Q9UMX0)	Protein linking IAP with cytoskeleton 1	n.d.	n.d.	n.d.	Heir et al. (2006)
NEDD8 (Q15843)	Ubiquitin-like protein Nedd8	Yes	JD	NEDD8	Ferro et al. (2007)
Parkin (O60260)	E3 ubiquitin-protein ligase parkin	Yes	JD, UIMs	IBR domain, Ubiquitin-like (Ubl) domain	Durcan et al. (2011, 2012)
Ubc7 (P62253)	Ubiquitin-conjugating enzyme E2 G1	Yes (transient interaction detected using cross-linking reagents)	n.d.	n.d.	Durcan et al. (2012)
p45 (P62195)	26S proteasome regulatory subunit 8	Yes	N-terminal atxn3 region (residues 1–133)	n.d.	Wang et al. (2007)
20S Proteasome (P25786, P25787, P25788, P25789, P28066, P60900, O14818, P20618, P49721, P49720, P28070, P28074, P28072, Q99436)	Proteasome subunits α types 1-7 and β types 1-7	n.d.	N-terminal atxn3 region (residues 1–150)	n.d.	Doss-Pepe et al. (2003)
CHIP (Q9UNE7)	E3 ubiquitin-protein ligase CHIP	Yes, kD (atxn3:CHIP) = 2.2 μM, kD (atxn3:Ub-CHIP) = 0.1 μM	Atxn3 C-terminus (residues 133–357)	CHIP N-terminus	Jana et al. (2005), Scaglione et al. (2011)
VCP/p97 (P55072)	Transitional endoplasmic reticulum ATPase	Yes	Residues 277–281 (includes arginine/lysine-rich NLS)	N domain, residues 1-199	Hirabayashi et al. (2001), Doss-Pepe et al. (2003), Matsumoto et al. (2004,?) Boeddrich et al. (2006), and Zhong and Pittman (2006)
E4B (O95155)	Ubiquitin conjugation factor E4 B	Yes (with 79Q-ataxin-3)	n.d.	n.d.	Matsumoto et al. (2004)
OTUB2 (Q96DC9)	Ubiquitin thioesterase OTUB2	n.d.	n.d.	n.d.	Sowa et al. (2009)
USP13 (Q92995)	Ubiquitin carboxyl-terminal hydrolase 13	n.d.	n.d.	n.d.	Sowa et al. (2009)
KCTD10 (Q9H3F6)	BTB/POZ domain-containing adapter for CUL3-mediated RhoA degradation protein 3	n.d.	n.d.	n.d.	Sowa et al. (2009)
Tubulin dimer (Q71U36/P68363)	Tubulin α-1A, Tubulin β-2B	Yes, kD (atxn3:tubulin) = 50–70 nM	JD	n.d.	Mazzucchelli et al. (2009)
Dynein (Q9Y6G9)	Cytoplasmic dynein 1 light intermediate chain 1	n.d.	n.d	n.d.	Burnett and Pittman (2005)
HDAC6 (Q9UBN7)	Histone deacetylase 6	n.d.	n.d.	n.d.	Burnett and Pittman (2005)
**TRANSCRIPTIONAL REGULATION**					
p300 (Q09472)	Histone acetyltransferase p300	Yes	PolyQ-containing C terminus of atxn3 (residues 288–354)	n.d.	Li et al. (2002)
CBP (Q92793)	cAMP-response-element binding protein (CREB)-binding protein	Yes	PolyQ-containing C terminus of atxn3 (residues 288–354)	n.d.	Li et al. (2002)
PCAF (Q92831)	p300/CREB-binding protein-associated factor: histone acetyltransferase KAT2B	Yes	PolyQ-containing C terminus of atxn3 (residues 288–354)	n.d.	Li et al. (2002)
Histone H3/H4 (P68431/P62805)	Histone	Yes	JD + UIM1 and 2 (residues 1–288)	n.d.	Li et al. (2002)
HDAC3 (O15379)	histone deacetylase 3	Yes	n.d.	n.d.	Evert et al. (2006)
NCOR1 (O75376)	Nuclear receptor corepressor 1	n.d.	n.d.	n.d.	Evert et al. (2006)
MAML3 (Q96JK9)	Mastermind-like protein 3	n.d.	n.d.	n.d.	Ravasi et al. (2010)
EWSR1 (Q01844)	RNA-binding protein EWS	n.d.	n.d.		Vinayagam et al. (2011)
**SIGNAL TRANSDUCTION**					
CK2 (P19784)	Casein kinase II subunit α	Yes	n.d.	n.d.	Tao et al. (2008), Mueller et al. (2009)
GSK3B (P49841)	Glycogen synthase kinase-3 β	Yes	n.d	n.d	Fei et al. (2007), Vinayagam et al. (2011)
DNM2 (P50570)	Dynamin-2	n.d.	n.d.	n.d.	Vinayagam et al. (2011)
CDKN1A (P38936)	Cyclin-dependent kinase inhibitor 1	n.d.	n.d.	n.d.	Vinayagam et al. (2011)
ANXA7 (P20073)	Annexin A7	n.d.	n.d.	n.d.	Vinayagam et al. (2011)
RPS6AK1 (Q15418)	Ribosomal protein S6 kinase α-1	n.d.	n.d.	n.d.	Vinayagam et al. (2011)
TK1 (P04183)	Thymidine kinase, cytosolic	n.d.	n.d.	n.d.	Vinayagam et al. (2011)
MKNK1 (Q9BUB5)	MAP kinase-interacting serine/threonine-protein kinase 1	n.d.	n.d.	n.d.	Vinayagam et al. (2011)
**ATAXIOME**					
TEX11 (Q8IYF3)	Testis-expressed sequence 11 protein	n.d.	n.d.	n.d.	Lim et al. (2006)
C16orf70 (Q9BSU1)	UPF0183 protein C16orf70	n.d.	n.d.	n.d.	Lim et al. (2006)
ARHGAP19 (Q14CB8)	Rho GTPase-activating protein 19	n.d.	n.d.	n.d.	Lim et al. (2006)
PICK1 (Q9NRD5)	PRKCA-binding protein	n.d.	n.d.	n.d.	Lim et al. (2006)

*Boxes shaded in gray represent associations identified in high-throughput interactome screenings*.

*Atxn3, ataxin-3; IBR, In Between Ring fingers; JD, Josephin domain; n. d., not determined; NLS, nuclear localization sequence; Ub, ubiquitin; UBA, ubiquitin associated domain; Ubl, ubiquitin-like domain; UIM, ubiquitin-interacting motifs*.

#### Ataxin-3 molecular function: ubiquitin hydrolase

A role for ataxin-3 in ubiquitin-dependent pathways was proposed by bioinformatic analysis (Scheel et al., 2003; Albrecht et al., 2004), and its ability to bind and cleave poly-ubiquitin chains and polyubiquitinated proteins was later demonstrated experimentally (Burnett et al., 2003; Chai et al., 2004). Importantly, inhibition of ataxin-3 catalytic activity results in the increase of polyubiquitinated proteins, resembling the effects of proteasome inhibition (Berke et al., 2005), indicating that ataxin-3 is involved with proteins targeted for proteasomal degradation. The function of ataxin-3 in the ubiquitin-proteasome system was further supported by the identification of its association with the ubiquitin-like domain of the human homologs of the yeast DNA repair protein Rad23, HHR23A, and HHR23B (Wang et al., 2000; Doss-Pepe et al., 2003; Nicastro et al., 2005, 2009), with valosin-containing protein (VCP)/p97 (Hirabayashi et al., 2001; Doss-Pepe et al., 2003; Boeddrich et al., 2006; Zhong and Pittman, 2006), and with the ubiquitin ligase E4B (Matsumoto et al., 2004) (Figures 4 and 5). Strikingly, the weak direct association between ataxin-3 and E4B is strongly reinforced by the addition of VCP/p97, indicating that these proteins form part of a higher order macromolecular complex to regulate the degradation of misfolded ER proteins (Matsumoto et al., 2004; Zhong and Pittman, 2006) (Figure 5).

Biochemical studies showed that ataxin-3 displays a strong preference for chains containing four or more ubiquitins (Chai et al., 2004) and that full-length ataxin-3 and its JD both display proteolytic activity toward either linear substrates containing a single ubiquitin molecule (Burnett et al., 2003; Chow et al., 2004b; Weeks et al., 2011) or K48/K63-linked poly-ubiquitin chains (Winborn et al., 2008; Todi et al., 2009), displaying also the capacity to bind the ubiquitin-like protein NEED8 in a substrate-like fashion (Ferro et al., 2007). Moreover, ataxin-3-like protein, Josephin 1 and Josephin 2, also display ubiquitin protease activity (Tzvetkov and Breuer, 2007; Weeks et al., 2011), although the relative activities are highly variable in spite of their high sequence similarity. Characterization of ataxin-3 ubiquitin hydrolase activity has also revealed that the full-length protein preferentially cleaves Lys-63-linked and mixed-linkage chains with more than four ubiquitins (Burnett et al., 2003; Winborn et al., 2008). This specificity is dictated by the UIMs, as the isolated JD shows a preference toward the disassembly of Lys-48-linked chains (Nicastro et al., 2009, 2010). Altogether, this indicates that ataxin-3 ubiquitin hydrolase activity is likely to be associated with delivery of target substrates to the proteasome rather than with their rescue from degradation, as it happens with most of the other deubiquitinases (Ventii and Wilkinson, 2008; Matos et al., 2011; Scaglione et al., 2011). Interestingly, ubiquitin hydrolase activity of ataxin-3 is not affected by polyQ expansion and both normal and expanded ataxin-3 are able to increase the cellular levels of a short-lived GFP normally degraded by the ubiquitin-proteasome pathway (Burnett et al., 2003).

The 3D structures for JD alone or in the presence of ubiquitin as well as that of the tandem UIM1-UIM2 have already been determined (Mao et al., 2005; Nicastro et al., 2005, 2009; Song et al., 2010), giving a structural perspective on the ubiquitin hydrolase function of ataxin-3. The JD contains two ubiquitin binding sites, both of hydrophobic nature, with site 1 being negatively charged to facilitate docking of the positively charged ubiquitin C-terminus close to the catalytic site. Binding of ubiquitin to site 1 is of crucial importance for both JD and full-length ataxin-3 activity as ubiquitin hydrolase (Nicastro et al., 2010). Site 2 confers ubiquitin-chain linkage preference to ataxin-3 and it overlaps with the surface for interaction of the ubiquitin-like domain in HHR23B (Nicastro et al., 2005, 2010). Solution structure for the two UIMs (UIM1 and UIM2), which are separated by a short 2 amino acid spacer, revealed that they fold into two α-helices separated by a flexible linker (Song et al., 2010). Upon ubiquitin binding, this structure adopts a typical helix-loop-helix folding pattern, where hydrophobic interactions dominate the complex formation (Song et al., 2010). When in tandem, UIM1 and UIM2 show higher binding affinity for mono- or poly-ubiquitin than individual UIMs (Song et al., 2010), suggesting a cooperative binding mechanism (Song et al., 2010). The effect of the presence of UIM3 in ataxin-3 binding affinity for ubiquitin has not been shown, but its role in ubiquitin chain binding and recognition is unlikely to be of relevance to ataxin-3 activity, since no differences in proteolytic activity were identified when the 2UIM and 3UIM isoforms were compared. In the model proposed for ataxin-3 ubiquitin chain proteolysis, the UIMs (UIM1-UIM2) select and recruit poly-ubiquitin substrates, presenting them to the catalytic JD for cleavage (Mao et al., 2005).

Even though ataxin-3 functions as ubiquitin hydrolase, its proteolytic activity is rather low, indicating that either ataxin-3/JD requires additional factors (post-translational modifications, cofactors, intracellular interactions) to exhibit significant proteolytic activity or the substrates used *in vitro* so far are not optimal. Interestingly, only three amino acid mutations are sufficient to significantly increase the proteolytic activity of ataxin-3, to a value close to that of ataxin-3-like protein (Weeks et al., 2011). Under physiological conditions, one candidate for an activating signal is mono-ubiquitination at K117, which has been shown to increase the enzyme’s rate of cleavage of Lys-63 linked substrates (Todi et al., 2009). However, the molecular mechanism by which ubiquitination increases enzyme activity is not still clear, nor is it known whether other cellular signals (e.g., phosphorylation by CK2 or GSK3b; Fei et al., 2007; Tao et al., 2008) may also modulate the activity of ataxin-3. Interestingly the JD-containing protein, Josephin 1 was also demonstrated to cleave ubiquitin chains only after it is mono-ubiquitinated (Seki et al., 2013). The regulation of ataxin-3 activity through ubiquitination might depend on the interaction of ataxin-3 with several E3 ubiquitin ligases (Durcan and Fon, 2013), such as the C-terminus of 70 kDa heat-shock protein (Hsp70)-interacting protein (CHIP), parkin, and E4B (Figure 5), since all were shown to promote ataxin-3 ubiquitination and regulate its degradation by the proteasome (Matsumoto et al., 2004; Jana et al., 2005; Miller et al., 2005). Association of ataxin-3 with CHIP is a multistep process regulated by mono-ubiquitination of the N-terminal region of CHIP by the E2-conjugating enzyme Ube2w, and occurs through the region encompassing polyQ and UIM1 and 2 (Jana et al., 2005) (Figure 4). As observed for other interactions involving the C-terminal region of ataxin-3, the ataxin-3-CHIP complex is affected by polyQ expansion and the polyQ-expanded protein displays a sixfold increase in binding affinity (Scaglione et al., 2011). The presence of ataxin-3 in multicomponent E3-ligase complexes is also supported by the identification of a direct interaction with parkin, an association that stabilizes the interaction between parkin and the E2-conjugating enzyme Ubc7 (Durcan et al., 2011). In contrast with what is observed in the ataxin-3:CHIP complex, ataxin-3 association with parkin remains unaltered by polyQ expansion (Durcan et al., 2012) (Figure 4). However, we still do not understand the mechanisms that regulate shuttling of ataxin-3 between these functional complexes or how its distribution is modulated by polyQ expansion. Further biochemical studies are required to establish the correlation between these macromolecular interactions and their relevance for ataxin-3 aggregation and neurodegeneration in MJD patients

### Ataxin-3 aggregation: A multistep pathway modulated by the protein context

A characteristic hallmark of MJD and other polyQ-expansion diseases is the appearance of intracellular inclusions enriched in the disease protein and containing components from the cell-quality control machinery (e.g., ubiquitin, proteasome subunits, and chaperones), indicating that these diseases form part of the larger family of protein misfolding disorders (Williams and Paulson, 2008). Early *in vitro* studies showed that expansion of the polyQ tract within the pathological range induced formation of insoluble β-rich fibrils with the capacity to bind amyloid-specific dyes (Bevivino and Loll, 2001). Later it was demonstrated that non-pathological ataxin-3 could also form insoluble fibrillar aggregates upon destabilization of its structure by temperature, pressure or denaturing agents (Marchal et al., 2003; Chow et al., 2004c). Unexpectedly, under partially destabilizing conditions the JD was also able to form insoluble fibrils, indicating that even though polyQ repeats are themselves toxic, the structure of ataxin-3, besides polyQ, has an important role in aggregation and fibril formation (Masino et al., 2004). Structural studies have shown that in ataxin-3, the addition of a polyQ tract destabilized the protein but polyQ expansion within the pathogenic range did not further induce significant structural changes (Chow et al., 2004a). In fact, alterations in ataxin-3 stability were later shown not to be a requirement for amyloid fibril formation since both non-expanded ataxin-3 and the isolated JD were shown to self-assemble and form fibrils under near-physiological conditions (Gales et al., 2005). Since kinetics of aggregation is slower for non-expanded ataxin-3 (Ellisdon et al., 2007), early events in the oligomerization pathway could be identified, with the formation of JD-mediated dimers proposed to be the first step in the ataxin-3 self-assembly pathway (Gales et al., 2005; Masino et al., 2011; Scarff et al., 2012). In a striking parallel with the classical amyloids the kinetics of fibril formation follows a nucleation-dependent polymerization mechanism, where these intermediate species might represent the most toxic species (Kayed et al., 2003; Laganowsky et al., 2012).

The current model for polyQ-expanded ataxin-3 aggregation consists of two steps. A JD-dependent step that leads to the formation of SDS-soluble protofibrils, followed by the formation of detergent-resistant fibrils similar to those found in MJD, where the polyQ-expanded tracts play a key role (Ellisdon et al., 2006, 2007). On the contrary, non-expanded polyQ ataxin-3 undergoes a single step aggregation event resulting in SDS-soluble aggregates, which does not involve the polyQ tract (Ellisdon et al., 2006) but directly depends on conformational changes outside the polyQ repeat. In the initial stages of aggregation, JD retains a native-like secondary structure, but is deployed of catalytic activity pointing to a subtle conformational change before fibril assembly (Masino et al., 2011). Although the JD plays a central role in this aggregation process, recent studies show that the ataxin-3 flexible region (Masino et al., 2003; Scarff et al., 2012), encompassing UIM1, and UIM2 also contributes to aggregation of the full-length protein (Santambrogio et al., 2012). This multistep aggregation modulated by the polyQ protein context seems to be common in other polyQ disorders such as HD (Thakur et al., 2009) and SCA1 (de Chiara et al., 2005). The presence of an expanded polyQ tract leads subsequently to rapid inter-fibril association and formation of large, highly stable amyloid-like fibrils. This indicates that the non-polyQ domains predispose ataxin-3 for aggregation and that the presence of a pathological polyQ tract introduces an additional step resulting in the formation of a highly stable amyloid-like aggregate.

This complex aggregation mechanism, involving domains outside the amino acid-repeat region, is also found in PABPN1(Tavanez et al., 2005; Winter et al., 2013), a multi-domain polyA-containing protein consisting of an N-terminal segment containing the alanine repeat, a coiled-coil domain, a RNA recognition motif (RRM), and a C-terminal domain. As observed for ataxin-3, the propensity of PABPN1 to aggregate and form amyloid fibrils (Scheuermann et al., 2003) is not exclusive of the expanded protein and can also be observed in the non-disease protein (Tavanez et al., 2005; Rohrberg et al., 2008). This indicates that polyA-flanking regions/domains influence the aggregation process, and that this is likely a broader mechanism common in homopeptide repeat-containing proteins. In fact, analysis of PABPN1 sequence indicates higher scores for aggregation propensity within the RRM domain (Tavanez et al., 2009), with mutations in the RRM being sufficient to prevent aggregate formation (Tavanez et al., 2005).

Aggregation of homopeptide repeat-containing proteins is therefore a multiparametric process that culminates in cell-specific degeneration, whose toxicity might be explained by the context-dependent molecular interactions and post-translational modifications. The relation between protein interactions, function, and aggregation will be discussed below, with a particular focus on the polyQ-containing protein, ataxin-3.

### Ataxin-3 function vs. aggregation

Several studies have been focused on the search for specific ataxin-3 interactors, some of which have identified direct physical association between the protein partners and provided clues into ataxin-3 biological role (Table 2 and references herein; Figure 5). Recently, different high throughput interactome screens focused on the search for protein complexes associated with ubiquitin hydrolases (Sowa et al., 2009), ataxia-related proteins (Lim et al., 2006), signal transduction pathways (Vinayagam et al., 2011), and transcriptional regulation (Ravasi et al., 2010), contributing with data on novel putative ataxin-3 binding proteins (Table 2; Figures 4 and 5).

An overview of the current data on the ataxin-3 interactome shows that a large number of interactions map to the catalytic JD. The intrinsic tendency of JD to self-associate involves the hydrophobic patches on its surface, which overlap with the functionally relevant ubiquitin binding sites 1 and 2 (Matos et al., 2011; Pastore and Temussi, 2012), providing a direct link between protein function and aggregation and exposing a role for intracellular interactors, such as ubiquitin, in protecting against ataxin-3 self-assembly (Gales et al., 2005; Masino et al., 2011; Matos et al., 2011; Pastore and Temussi, 2012). Likewise, in the polyA-containing protein PABPN1 the RRM domain responsible for the interaction with the mRNA (Banerjee et al., 2013) and with polyadenylate-specific RNA polymerase is also involved in the aggregation process (Winter et al., 2013). In addition, the fact that heat-shock proteins including Hsp70, and type I arginine methyl transferases (PRMT1 and PRMT3) associate preferentially with expanded PABPN1 raises the question whereas proteotoxicity of expanded PABPN1 might also be caused by altered protein networking. Destabilization of the ataxin-3 JD by specific mutations revealed that any conformational change in this region is directly linked with aggregation of the full-length protein (Saunders et al., 2011), emphasizing the putative therapeutical potential associated with the identification of macro-molecules with the ability to stabilize this N-terminal region. In fact, interaction of JD with protein partners (Masino et al., 2011) or chaperones (Robertson et al., 2010) is sufficient to induce stabilization of JD leading to a reduction in ataxin-3 self-assembling properties (Matos et al., 2011; Pastore and Temussi, 2012).

Concerning the C-terminal flexible tail of ataxin-3, the interaction with VCP/p97 is probably the one for which more experimental data is available (Hirabayashi et al., 2001; Doss-Pepe et al., 2003; Matsumoto et al., 2004; Boeddrich et al., 2006; Zhong and Pittman, 2006). This association is dependent on the arginine/lysine-rich motifs close to the polyQ tract of ataxin-3 (Boeddrich et al., 2006), and several reports point to a stronger interaction with the disease-protein containing longer polyQ stretches (Wang et al., 2000; Matsumoto et al., 2004; Boeddrich et al., 2006; Zhong and Pittman, 2006). Functional interaction with VCP/p97 is able to modulate the fibrillogenesis of a C-terminal fragment of expanded ataxin-3 (71Q) in a concentration-dependent manner, with equimolar concentrations of VCP/p97 stimulating fibrillogenesis, while a fourfold excess of VCP prevented aggregation (Boeddrich et al., 2006). This study provides clues toward the role of interactors targeting the C-terminal region of ataxin-3 as modulators of its oligomerization properties, indicating that this mostly unstructured region (Masino et al., 2003; Scarff et al., 2012) may also represent a bridge between physiological interactions, function, and aggregation. In the field of polyQ disorders, the search for protein interactors is an active area of research, uncovering novel macromolecular partners often acting as disease modifiers (Goehler et al., 2004; Kaltenbach et al., 2007; McGurk and Bonini, 2012). A recent screen for modifiers of ataxin-1 aggregation and toxicity in mammalian cells showed that toxicity enhancers often contained coiled-coil domains. Importantly, coiled-coil formation by ataxin-3 polyQ stretch and its flanking domains were also predicted (Fiumara et al., 2010), however neither the consequences of its expansion nor its functional properties were experimentally assessed. Since coiled-coil structures are known to be involved in protein–protein interactions (Parry et al., 2008; Fiumara et al., 2010), it would be interesting to access the role of protein interactions mediated by the polyQ region of ataxin-3 in regulating its aggregation behavior (Figure 4). In fact, some of the interactors associated with ataxin-3 nuclear functions, rich in polyQ- (p300, NCOR, CBP), and polyA-repeats (NCOR), target this region and are predicted to bind better to the expanded protein (Figures 4 and 5). Therefore it is tempting to speculate that these anomalously stronger interactions with the expanded protein in the nucleus might be associated with increased aggregation and toxicity observed when a strong nuclear localization sequence (NLS) is fused to ataxin-3 (Perez et al., 1998; Bichelmeier et al., 2007; Macedo-Ribeiro et al., 2009) or when the protein shuttles to the nucleus upon increased cellular stress (Reina et al., 2010). Heat-shock induces phosphorylation of a serine residue located on ataxin-3 JD and drives the protein to the nucleus leading to its dissociation from the cytosolic VCP/HHR23A complex (Reina et al., 2010), highlighting the dynamic nature of ataxin-3 partition between macromolecular assemblies and making it tempting to speculate that polyQ expansion might affect this distribution.

## Concluding Remarks

Trinucleotide repeats are keen for driving evolution by providing genetic variability, with homopeptide-encoded regions being crucial for the establishment of protein interactions. However, as unstable regions, expansion of the homopeptide regions might occur, being responsible for several neurodegenerative and muscular diseases. Homopeptide repeats such as polyQ and polyA seem to influence and to drive the repeat-containing protein toward self-assembly and aggregation. On the other hand, structural studies were able to reveal that aggregation of homopeptide-containing proteins also depends on the homopeptide protein context with additional protein domains playing a role in a multi-domain self-assembly mechanism. Differential expression of protein isoforms generated by alternative splicing, post-translational modifications and, additionally, differences in macromolecular interactions are currently advanced as hypotheses that, by their ability to modulate protein function and aggregation, could explain the cell-specific toxicity of the homopeptide-expanded proteins.

Ataxin-3 is an excellent example of a repeat-containing protein that, upon polyQ-expansion, does not undergo drastic structural and functional changes, but achieves an increased tendency toward self-assembly and aggregation. The protein has deubiquitinase activity and plays a role in the cell-quality control system, and in transcriptional regulation. As a result of its modular multi-domain structure, ataxin-3 engages in multiple macromolecular interactions and several evidences show that it associates with several functional multiprotein complexes, in some cases in a polyQ-dependent manner. The structural and mechanistic details regulating ataxin-3 redistribution between different cellular machineries are still unclear, although post-translational modifications of the protein subunits assembled in these complexes are likely to play a role. Different cellular events such as proteotoxic stress or aging might unbalance the association of expanded ataxin-3 with its molecular partners and contribute to the alteration of ataxin-3 normal cellular functions. Since ataxin-3 self-assembly is a complex process that involves several protein domains, including JD, the relocalization of the protein to different complexes might induce the exposure of aggregation-prone regions and lead to the appearance of the characteristic intracellular protein inclusions.

Since macromolecular interactions seem to be either protective or exacerbate aggregation of the homopeptide-containing proteins, they might be targeted therapeutically. However, an in-depth understanding about the effect of homopeptide-expansion in the function of the containing-protein and on the interactions with molecular partners is required, in order to understand how they contribute to neurodegeneration. The combination of biochemical and computational approaches to the identification of disease-protein interaction networks is critical for defining their normal function, identifying new markers for disease prognosis and also for the development of tools to selectively target those interactions with potentially reduced side effects.

## Conflict of Interest Statement

The authors declare that the research was conducted in the absence of any commercial or financial relationships that could be construed as a potential conflict of interest.
